# Strain specific properties of *Escherichia coli* can prevent non-canonical amino acid misincorporation caused by scale-related process heterogeneities

**DOI:** 10.1186/s12934-022-01895-1

**Published:** 2022-08-23

**Authors:** Florian Mayer, Monika Cserjan-Puschmann, Benedikt Haslinger, Anton Shpylovyi, Thomas Dalik, Christian Sam, Rainer Hahn, Gerald Striedner

**Affiliations:** 1 Christian Doppler Laboratory for Production of Next-Level Biopharmaceuticals in E. Coli, Department of Biotechnology, Institute of Bioprocess Science and Engineering, University of Natural Resources and Life Sciences, Muthgasse 18, 1190 Vienna, Austria; 2 Department of Chemistry, Institute of Biochemistry, University of Natural Resources and Life Sciences, Muthgasse 18, 1190 Vienna, Austria; 3 Boehringer Ingelheim RCV GmbH & Co KG, Dr. Boehringer-Gasse 5-11, 1120 Vienna, Austria

**Keywords:** Scale-down, Recombinant protein production, Fab, Norleucine misincorporation, Escherichia coli

## Abstract

**Background:**

*Escherichia coli* is one of the most important hosts for production of recombinant proteins in biopharmaceutical industry. However, when selecting a suitable production strain, it is often not considered that a lot of different sub-species exist, which can differ in their genotypes and phenotypes. Another important development step is the scale-up of bioprocesses with the particular challenge that heterogeneities and gradients occur at production scale. These in turn can affect the production organism and can have negative impact on the process and the product quality. Therefore, researchers developed scale-down reactors, which are used to mimic manufacturing conditions in laboratory scale. The main objectives of this study were to determine the extent to which scale-related process inhomogeneities affect the misincorporation of non-canonical amino acids into the recombinant target protein, which is an important quality attribute, and whether strain specific properties may have an impact.

**Results:**

We investigated two industrially relevant *E. coli* strains, BL21(DE3) and HMS174(DE3), which produced an antigen binding fragment (Fab). The cells were cultivated in high cell density fed-batch mode at laboratory scale and under scale-down conditions. We demonstrated that the two host strains differ significantly with respect to norleucine misincorporation into the target protein, especially under heterogeneous cultivation conditions in the scale-down reactor. No norleucine misincorporation was observed in *E. coli* BL21(DE3) for either cultivation condition. In contrast, norleucine incorporation into HMS174(DE3) was already detectable in the reference process and increased dramatically in scale-down experiments. Norleucine incorporation was not random and certain positions were preferred over others, even though only a single codon exists. Differences in biomass and Fab production between the strains during scale-down cultivations could be observed as well.

**Conclusions:**

This study has shown that *E. coli* BL21(DE3) is much more robust to scale-up effects in terms of norleucine misincorporation than the K12 strain tested. In this respect, BL21(DE3) enables better transferability of results at different scales, simplifies process implementation at production scale, and helps to meet regulatory quality guidelines defined for biopharmaceutical manufacturing.

**Supplementary Information:**

The online version contains supplementary material available at 10.1186/s12934-022-01895-1.

## Background

About 30% of all recombinant therapeutic proteins were produced in *Escherichia coli* in the beginning of the last decade, and new approvals are still made today [1, 2], demonstrating the importance of this production host for the biopharmaceutical industry. The advantages of *E. coli* as robust, fast growing and well characterized host are well known in the scientific community [2], but the diversity of clones available in nature and industry is often overlooked. For example, some subspecies can induce severe health problems by production of toxins [3], while others are classified as biosafety level 1 and do not pose a severe health risk [4]. The different genotypes and resulting phenotypes of industry relevant strains were investigated in several studies [5–9].

In large-scale production processes of several m^3^, heterogeneities of substrate, dissolved oxygen (DO) and pH almost inevitably occur due to higher mixing times in the large-scale tanks [10–12]. These resulting gradients can affect the production organisms and thus influence the overall process by decreasing the biomass yield or increasing the byproduct formation [11, 13]. Moreover, even the protein of interest (POI) itself can be changed by the unwanted incorporation of non-canonical amino acids (ncAA) caused by the heterogenous conditions.

It was reported that norleucine, norvaline and β-methyl-norleucine can get misincorporated for methionine, leucine and isoleucine, respectively [14]. One proposed mechanism, illustrated by Reitz et al*.* [14], is based on an increased flux through the branched-chain amino acid pathway. This flux change is caused by the overflow metabolism and pyruvate accumulation in the cell originating from the heterogeneities the cells are exposed to, and by the intracellular amino acid depletion due to the high expression of the POI [15, 16]. Thereby, not only the production of canonical amino acids is increased, but also the production of the before mentioned ncAA. The higher intracellular level of ncAAs increases the probability of erroneous loading of the corresponding tRNAs, which results in the misincorporation into the POI [17]. Proteins high in branched chain amino acids, especially leucine, were reported to be prone to misincorporation, such as the biopharmaceutical relevant proteins Interleukin-2 [18], bovine somatotropin [15] and recombinant hemoglobin [19]. The amino acid sequence of biotechnological products is an important specification criterium for authorities as the European Medicines Agency [20]. The industrial relevance of this problem is further highlighted by several patents protecting methods to avoid the misincorporation by amino acid supplementation to the medium [21] or molecular biological changes of the host [22].

To simulate and investigate the scale-effects originating from heterogenous conditions in large-scales, researchers developed scale-down setups [23]. These systems have laboratory scale and consist of one or more compartments in which the production cells are exposed to different gradients intended to mimic industrial production conditions [24–26].

In this study, we used the scale-down setup described by Mayer et al*.* [27] to compare the process performance of the two different industrial relevant *E. coli* strains, BL21(DE3) and HMS174(DE3) [2]. These two strains were chosen due to the genotypic difference of K12 and B strains regarding the regulation of glucose uptake and metabolism described in literature [5, 28]. The scale-down setup was used to generate heterogeneities during the process and therefore, simulate a larger scale (m^3^). An antigen binding fragment (Fab) was chosen as model protein, because of its relevance for industry as biopharmaceutical [29, 30]. Due to the variability of the different sub-species, we hypothesized that the use of HMS174(DE3) and BL21(DE3) will reveal differences in the amount of the incorporated ncAA. Additionally, we used mass spectrometry to investigate, whether some methionine positions are favored for norleucine incorporation, although only one codon exists for this amino acid for translation [14].

## Results

### Scale-down compared to laboratory scale cultivations

To generate heterogeneities and gradients, usually occurring at production scale, a scale-down setup was used. We compared the overall performance of scale-down cultivations, using a two-compartment setup consisting of a stirred tank reactor (STR) combined with a plug-flow reactor (PFR), with lab-scale cultivations in a classical STR as reference. The two setups had the same size of 20 L and the only difference of the used processes were the connection of the plug-flow compartment at feed start. By generation of heterogeneities in the PFR of the scale-down setup, conditions similar to the ones in large-scale (m^3^ range) bioreactors were generated. Therefore, the results of the scale-down setup should mimic the outcome of a large-scale production process. The *E. coli* host strains BL21(DE3) and HMS174(DE3) producing the antibody fragment FTN2, named B < oFTN2 > and H < oFTN2 > , were grown in high cell density cultivations.

**Fig. 1:**
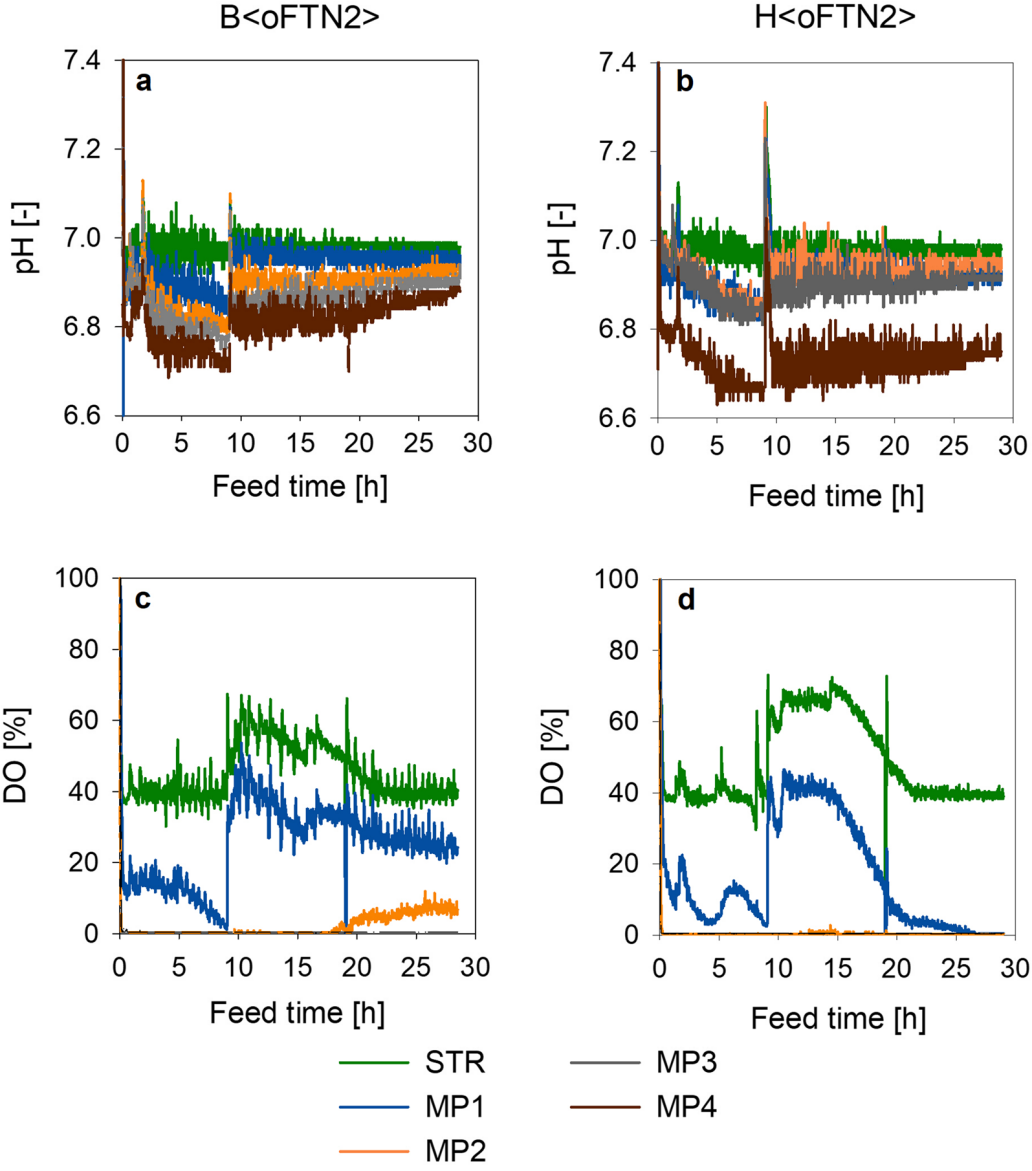
Online data from scale-down cultivations showing the pH and DO for B < oFTN2 > (**a**, **c**) as well as for H < oFTN2 > (**b**, **d**). The different measurement points (MP) correspond to following residence times (RT) in the PFR: MP1—5 s, MP2 – 13 s, MP3 – 20 s and MP4 – 33 s. For MPs with longer RT DO was at 0%

Online data for pH and DO from scale-down and reference cultivations (Fig. 1) already revealed differences between the strains. One measurement point (MP) was located in the STR and four MPs were analyzed along the PFR. The MPs in the PFR correspond to residence times (RT) of 5 s (sec), 13 s, 20 s and 33 s, respectively. For B < oFTN2 >, acidification along the PFR occurred faster at the beginning, but slowed down after longer RT in the PFR. The pH during H < oFTN2 > cultivations reached a plateau between MP1 to MP3 and then dropped from MP3 to MP4. Therefore, acidification during H < oFTN2 > cultivations was slower at smaller RT, but higher at the end of the PFR, compared to cultivations with B < oFTN2 > . Oxygen consumption rate was also different along the PFR between the two strains. For H < oFTN2 > , oxygen depletion was detected at MP2 over the whole course of the cultivation and even detected at MP1 at the end of cultivation, where the cell density was high. For B < oFTN2 >, oxygen consumption rate decreased in the second feed phase, 9 hours (h) after feed start. This decrease can be seen by the rise in DO at MP2.

**Fig. 2:**
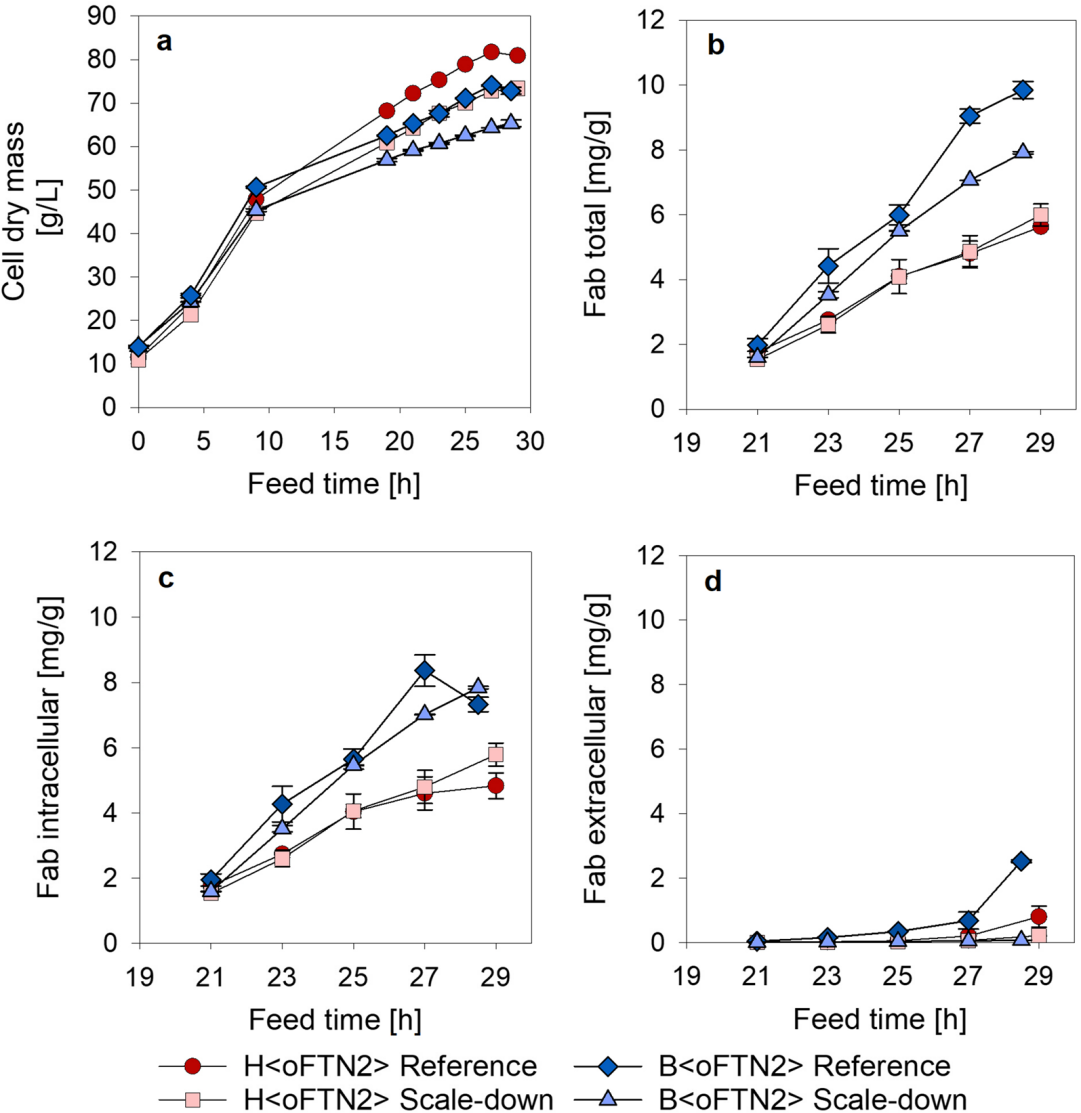
Biomass concentration (**a**) as well as total (**b**), intracellular (**c**) and extracellular (**d**) Fab production, given in mg Fab per g CDM for B < oFTN2 > and H < oFTN2 > . Data points show the average of biological duplicates, the error bars indicate the results of the two individual experiments

The biomass and Fab production for B < oFTN2 > , described by Mayer et al*.* [27], were used here for comparison to H < oFTN2 > . During the H < oFTN2 > reference cultivation (without PFR) 81 g/L cell dry mass (CDM) were reached (Fig. 2a), which is about 10% more biomass than with B < oFTN2 > . However, this difference in biomass yield was only observed in the second feed phase (growth rate (µ) = 0.05 h^−1^). In the first feed phase (µ = 0.17 h^−1^) B < oFTN2 > reached higher biomasses. The CDM reduction in scale-down cultivations compared to the reference process was about 9% for both strains.

The total specific Fab amount was almost the same with and without the scale-down setup for H < oFTN2 > (Fig. 2 b). Nevertheless, compared to the B < oFTN2 > reference cultivation, the decrease in Fab amount was a reduction of 40%. This loss in specific productivity could also not be compensated by the higher biomass yield of H < oFTN2 > , which still resulted in a 36% reduced production of Fab. Notable is that similar to B < oFTN2 > the intracellular Fab fraction was higher during the scale-down cultivations with H < oFTN2 > , even though not so pronounced (Fig. 2c and d). For the H < oFTN2 > reference cultivations, the extracellular Fab fraction was 14% whereas for the scale-down experiments it was 4%. Considering the intracellular Fab, the fraction in the inclusion body (IB) was neglectable compared to the soluble (cytosolic) fraction as can be seen in Additional file 1: Figure S1.

### Misincorporation of non-canonical amino acids

**Fig. 3:**
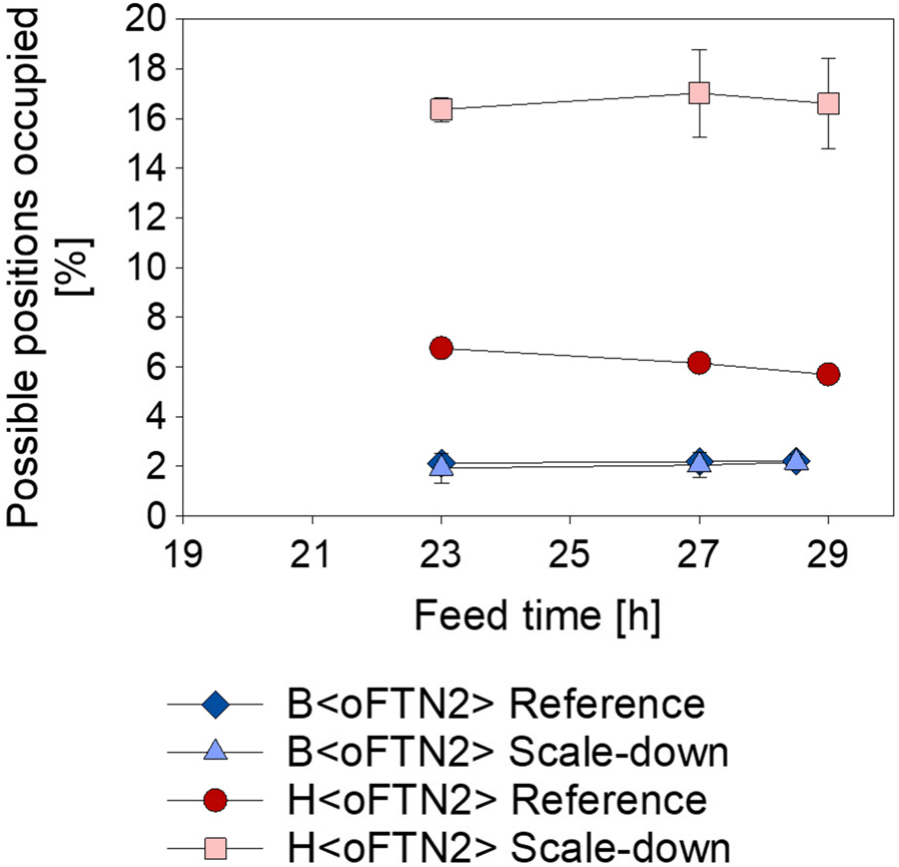
Norleucine misincorporation at the five methionine positions possible in the Fab FTN2. Data points show the average of biological duplicates, the error bars show the results of the individual experiments

To analyze the amount of misincorporated ncAA into the POI, the Fab was affinity purified, hydrolyzed and amino acids were quantified by high performance liquid chromatography (HPLC). β-methyl-norleucine was not targeted by this method and norvaline could not be quantified, because the concentrations in the samples were below the detection limit of the method. However, a higher incorporation of norleucine into the product was observed during cultivations with H < oFTN2 > (Fig. 3). The percentage of methionine positions occupied were constant over the course of the cultivations, but increased from approximately 6% to 17% when the cells were exposed to scale-effects in the PFR. Remarkably, during the cultivations with B < oFTN2 > only 2% of the possible incorporation sites were occupied with norleucine, even when the scale-down setup was used.

As norleucine could be quantified by the HPLC method, mass spectrometry (MS) was used to analyze if some methionine positions are more prone for misincorporation than others. Therefore, the purified Fab from the samples of H < oFTN2 > cultivated with the scale-down setup at the end of fermentation were digested with trypsin. This treatment led to five target fragments, each containing one of the methionine positions. Four methionines were located on the Fab heavy chain (HC), one was located on the Fab light chain (LC). Table 1 shows the results of the MS analysis given as a percentage of the abundances of fragments containing norleucine. Exemplarily shown, 18.44% of the fragment HC 1 contained norleucine while the remaining 81.56% contained the correct methionine. The average calculated in the last line of Table 1 shows how much norleucine was incorporated on average at all five positions. Therefore, this average gives the percentage of norleucine incorporation in the whole Fab molecule and can be interpreted as the random probability of norleucine misincorporation at any position. It can be seen, that the fragments HC1 and LC1 contained elevated values of norleucine compared to the other fragments, even though only one codon exists for methionine [14]. This method showed an average misincorporation of norleucine into the total Fab of approximately 14%, which is very comparable to the 16%- 17% obtained by the HPLC method. The significance of the observed differences described in Table 1 could not be tested by a statistical test, as only duplicates were measured. To give an estimate for the reproducibility of the individual experiments, the deviation of the two biological duplicates to the average is indicated. The deviations of single experiments to average were less than 2.6%, indicating high reproducibility.

**Table 1:** MS results for the percentage of Fab fragments found with norleucine

Fragment	H < oFTN2 > Scale-down 1 Norleucine [%]	H < oFTN2 > Scale-down 2 Norleucine [%]	Average Norleucine [%]	Deviation of single experiments to average [%]
HC 1	18.44	14.53	16.49	± 1.96
HC 2	13.69	8.50	11.10	± 2.60
HC 3	10.66	8.65	9.66	± 1.01
HC 4	14.35	11.25	12.8	± 1.55
LC 1	20.83	17.62	19.23	± 1.61
Average	15.59	12.11	13.85	± 1.74

The results for the biological duplicates of H < oFTN2 > cultivated with the scale-down setup at 29 h after feed start are given. Four fragments resulted from the Fab heavy chain (HC) and one from Fab light chain (LC). The column ‘Average Norleucine’ represents the average of the biological duplicates, the column ‘Deviation of single experiments to average’ gives the deviation of the biological duplicates to the average

## Discussion

The comparison of both *E. coli* strains revealed major differences in the process performance during the high cell density cultivations. H < oFTN2 > had a higher biomass yield, whereas B < oFTN2 > was the much better Fab producer. Those findings are in contradiction to Fink et al*.* [31], where exactly the opposite was found. Nevertheless, the difference most probably lies in the different media composition, the different induction strategy and µ used during the feed phase. These results show that the cultivation conditions have a big impact on the behavior of different *E. coli* strains during recombinant protein production and can even reverse existing results.

The 9% biomass reduction found for H < oFTN2 > , when using the scale-down setup, was almost the same as for B < oFTN2 > [27]. This reduction is in accordance with Bylund et al*.* [11], who reported a 15% biomass reduction in a 12 m^3^ bioreactor, when the feed was added directly at the reactor bottom to the medium. In the case of H < oFTN2 > , the total specific Fab production was not affected by the heterogenous conditions generated by the scale-down setup, which suggests that this strain is more robust than B < oFTN2 > in this respect. Nevertheless, as B < oFTN2 > had a much higher productivity even during the scale-down cultivations, it would be advisable to choose this strain for the Fab production process. Remarkably, also a higher extracellular Fab fraction was found during the reference cultivations with H < oFTN2 > as already described for B < oFTN2 > [27]. This finding is supported by other literature describing that cells become less permeable and therefore more viable when exposed to heterogenous conditions [32, 33]. For B < oFTN2 > , the higher viability of the cells during the scale-down cultivations could be shown by the lower extracellular DNA content at fermentation end (Additional file 1: Table S2), which is a sign for a lower amount of cell lysis. For H < oFTN2 > , no significant difference in extracellular DNA concentration between reference and scale-down cultivations could be observed. Therefore, the higher amount of extracellular Fab in H < oFTN2 > reference cultivations could not be explained by an increased amount of cell lysis. The observed increase in cell lysis during B < oFTN2 > reference cultivations and the distribution of the product between the intra- and extracellular compartment can complicate downstream processing. Therefore, an increase in cell membrane robustness and cell viability can be interpreted as a beneficial scale-effect. In this respect, it is also important to mention that the intracellular Fab fraction was produced soluble in the periplasm and was not aggregated into IB, which would make an additional refolding process necessary.

One of the most interesting findings of this study was that B < oFTN2 > did not show increased norleucine incorporation into the Fab product. This is in accordance with Ni et al*.* [34], who did not find ncAA incorporation into a recombinant protein vaccine candidate when they used BL21(DE3) during well-mixed laboratory scale cultivations. However, it is a novelty that we could show that the incorporation did not even take place, when the cells were exposed to the heterogenous conditions in our scale-down setup, which should favor the production of ncAA. The misincorporation for H < oFTN2 > could be interpreted as high, by keeping in mind that a value of 20% would mean that each Fab contains on average one norleucine, as the Fab contains five methionine positions. It is also notable to mention that the used Fab FTN2 is not rich in branched chained amino acids (leucine: 7.1%, valine: 8.5%, isoleucine: 2.5%) or methionine (1.1%), which should make it less prone for misincorporation [14].

These results highly suggest that BL21(DE3) and HMS174(DE3) have different strategies to cope with the effects induced by the scale-down setup. The online data shown in Fig. 1 further support this hypothesis by showing that H < oFTN2 > had an increased oxygen demand. Additionally, H < oFTN2 > released acidifying metabolites, like organic acids or carbon dioxide, after longer RT in the PFR and then to a higher extent than B < oFTN2 > .

Marisch et al*.* [5] reported that the gene for the outer membrane porin C (*ompC*), a porin responsible for carbon transport, is dysfunctional in BL21. Altered carbon transport into the cell could result in different amounts of pyruvate produced and therefore alter the flux to the branched chain amino acid pathway.

BL21 strains were reported to produce less acetate than K12 strains [35], which shows that the cells handle overflow metabolism in a different way. It is controversially discussed if this difference in acetate production originates from the use of the glyoxylate shunt in the tricarboxylic acid cycle [5, 28, 36]. However, as the cells are exposed to oxygen limitation in the PFR, a higher flux through the TCA cycle is in our opinion unlikely. Therefore, pyruvate produced in the cell has to be channeled to other pathways. Yoon et al*.* [7] reported that the biosynthesis of amino acids is upregulated in a B strain compared to a K12 strain. This increase in amino acid synthesis would have several advantages and could explain our findings. The cell would have an option to cope with the overflow metabolism induced by the scale effects and the higher intracellular levels of canonical amino acids would lower the chance of misincorporation of ncAA, due to affinity differences [17]. Additionally, the higher availability of amino acids could be beneficial for recombinant protein production [7]. Nevertheless, the comparability between the study of Yoon et al*.* and ours is limited. Yoon et al*.* used other K12 strains as described in our study. Additionally, the cells were cultivated in glucose-unlimited shake flask cultures, whereas we used carbon-limited fed-batch bioreactor cultivations.

In this study, no elevated values of incorporated norvaline were detected during scale-down experiments, whereas reports of norvaline incorporation exist in the literature [19]. However, according to Reitz et al. [14], in most cases either norleucine or norvaline incorporation were reported. This behavior could also be a strain- or protein-dependent issue.

Another outcome of the study was information about the positions where the norleucine incorporation occurred. As expected, it could be shown that misincorporation occurred at all methionine positions, as only one codon for methionine exists. Nevertheless, the probability to find norleucine was higher especially at the positions at the beginning of HC and LC. The fact that the incorporation is not completely random was reported also by other authors [37]. Possible explanations for this phenomenon could be differences in the adjacent codon context of the methionine codons, or the secondary structure of the growing protein chain, where it was described that incorporation is more likely in random coils than in defined structures [38].

## Conclusions

In this study, we clearly demonstrated that different *E. coli* strains react differently to heterogenous conditions induced by a scale-down setup. This difference was shown not only for biomass and recombinant protein production, but also for ncAA misincorporation into the product. Using the host strain BL21(DE3), this unwanted phenomenon was avoided even in scale-down cultivations, which should favor misincorporation. Another finding was that the probability for incorporation of the ncAA norleucine was not the same at each methionine position. Even if only one codon is used for translation, favored positions seem to exist. The authors are aware that experiments at large-scale (m^3^ range) would be beneficial to verify the results obtained from the scale-down setup. However, as a setup was used, which is commonly acknowledged by the scientific community to reproduce large-scale effects, this study describes a simple option to avoid ncAA misincorporation into recombinant proteins during process scale-up. By the use of more robust strains, e.g. BL21(DE3), no additional measures have to be taken, neither during the process, nor during strain engineering. This avoids the risk of violating existing patents protecting those methods and can help to provide safe biopharmaceuticals sticking to the authority’s guidelines after a fast scale-up.

## Methods

### *E. coli* reference and scale-down cultivations

To test the behavior of two different industrial relevant *E. coli* strains during scale-down experiments, the strains BL21(DE3) (New England Biolabs, USA) and HMS174(DE3) (Novagen, USA) were used. For recombinant protein production, the sequence of the Fab FTN2, specific for tumor necrosis factor α (TNFα), was genome-integrated together with an OmpA translocation sequence, to direct the Fab to the periplasm, as described by Fink et al*.* [39]. The resulting strains were called B < oFTN2 > and H < oFTN2 > respectively.

To investigate the influence of scale-effects on the process, a custom-build laboratory scale-down setup was used [27]. For that purpose, the scale-down setup was designed on results from computational fluid dynamics simulation of a lab-scale and a production scale bioreactor. We chose a two-compartment system consisting of STR (Bioengineering, Wald, Switzerland) and a PFR to generate the conditions of a m^3^ scale bioreactor in laboratory scale. Briefly, the STR had a maximum working volume of 20 L and the PFR a volume of 4.16 L. For the reference cultivations, only the 20 L STR was used, for the scale-down experiments, the PFR was connected at the beginning of feeding phase and the feeding solution was added at the beginning of the PFR. Apart from the connection of the PFR, the procedure of the reference and scale-down experiments was exactly the same. The strains were cultivated in carbon-limited fed-batch mode with glucose to high cell densities. Details about the used media, process characteristics and design of the scale-down setup are extensively described by Mayer et al*.* [27]. Briefly, the fermentation strategy consisted of two exponential growth phases with a µ of 0.17 h^−1^ for the first 9 h, followed by a second phase with a µ of 0.05 h-^1^ for another 20 h. The batch medium was a semi-synthetic medium containing yeast extract, where the salts and glucose were added according to the amount of biomass to be produced. The feed solution contained 54% (w/w) glucose as carbon source and had the same composition as the batch medium, except for yeast extract, PPG 2000 and NH_4_SO_4_, which were only added to the batch medium. The process was designed to produce 120 g CDM in 10 L medium until batch end. In the feed phase, the CDM should be increased to 1506 g CDM in approximately 18 L. The pH was set to 7 and the DO to 40%. Due to oxygen supply problems at the end of cultivations with B < oFTN2 > , all cultivations with this strain were stopped half an hour earlier. 19 h after feed start, induction was done with a single pulse of 1 µmol IPTG per g CDM calculated for the biomass at fermentation end.

### Biomass and product analysis

For biomass analysis, gravimetrical CDM und OD_600_ measurements were done according to Marisch et al*.* [6]. The sampling of cell pellets for intracellular product analysis was described by Fink et al*.* [31]. For extracellular Fab analysis, cell supernatant was stored at − 20 °C. The cell lysis protocol and the Fab quantification by a sandwich enzyme-linked immunosorbent assay (ELISA) was described in the literature previously [27, 39].

### Fab purification

To purify Fab for ncAA analysis, 45 mL cell suspension were sampled at 23 h and 27 h after feed start as well as at fermentation end. The suspension was centrifuged, the supernatant was discarded and the pellets were stored at − 20 °C. For Fab extraction the cells were resuspended in buffer containing 100 mM Tris and 10 mM EDTA at pH 8, to approximately 22% (w/w). 1.5 mL of the suspension were aliquoted and the samples were incubated for 1 h at 60 °C at a shaking frequency of 400 rpm (Thermomixer comfort, Eppendorf, Germany). Afterwards the suspension was centrifuged for 20 min at 21,000 rcf. The supernatant was stored and filtered with a 0.22 µm syringe filter. 12 mL of the extract were used for purification with ProteinG affinity chromatography.

An ÄKTA™ pure workstation (Cytiva, Sweden) was used in combination with a KanCap™ G (Kaneka, Japan) prepacked column. Sample load was done via the sample pump and elution was done with a step elution. Detailed information about the used buffers, as well as the purification protocol is given by Schimek et al. [40]. An exemplary chromatogram can be found in Additional file 1: Figure S2a. The eluted purified Fab solution was stored at 4 °C and used for ncAA quantification by HPLC.

For the samples taken at fermentation end, additional purifications were performed. During these runs the eluted Fab solution after the affinity purification was collected in a sample loop and loaded on a Source 15S column (Cytiva, Sweden) for cation exchange (CIEX) chromatography. This step was used to further analyze the purified Fab. The used buffers and chromatography protocol can be found in literature [40]. A chromatogram can be found in Additional file 1: Figure S2b. The resulting peak during the elution step was fractioned and analyzed by MS.

### Fab hydrolysis and non-canonical amino acid analysis using HPLC

Prior to ncAA amino acid quantification the affinity purified Fab had to be hydrolyzed to obtain the free amino acids. Therefore, aliquots containing approximately 10 µg Fab were transferred to 1 mL glass ampoules (SCHOTT, Germany). 10 nmol of taurine (Sigma-Aldrich, MO, USA) were added as internal standard and the solution was dried by using a Savant™ SPD131DDA SpeedVac™ concentrator (Thermo Fisher Scientific, MA, USA) at 45 °C. After this step 100 µL of a solution containing 6 M HCl, 0.1% Phenol and 0.1% Thioglycolic acid were added. A centrifuge was used to spin down the solution and hydrolysis was started for 24 h, at 100 °C under vacuum. The solution was again dried and the hydrolyzed Fab was dissolved in 100 µL high-quality water (Ultra Clear Basic, SG, Germany). The sample was transferred to a HPLC vial and was used for further analysis.

For amino acid separation and quantification, a Series 1050 HPLC (Hewlett Packard/Agilent Technologies, CA, USA) was used together with a Kinetex^®^ 5 µm C18 100 Å, 250 × 4.6 mm, liquid chromatography column (Phenomenex, CA, USA). The samples were precolumn derivatized by using 4 µL o-Phthaldialdehyde dissolved in 0.4 M sodium borate buffer pH 10.0 containing 1% Brij 35 and 0.4% 2-mercaptoethanol [41]. For the separation 100 mM Na-acetate buffer pH 6.6 with 1% Tetrahydrofuran and 0.001% Na-azide was used as solvent (A). The elution buffer (B) was a solution containing 50% 35 mM Na-acetate pH 6.6 and 50% Acetonitrile. A gradient from 20% to 57% B for 25 min was used and followed by a second gradient from 57% to 95% B for 4 min. Afterwards 100% Acetonitrile was applied for 2 min followed by 4 min of 25% B to regenerate the column. The flow rate was 1.2 mL/min during the whole process and the pressure was at 180 bar. For detection a RF-10AXL fluorescence detector (Shimadzu, Japan) at an excitation wavelength of 340 nm and an emission wavelength of 450 nm was used. Elution profiles can be seen in Additional file 1: Figure S3.

The peak area of the internal taurine standard was used together with empirical correction factors (Additional file 1: Table S3) and the peak areas of the different amino acids to calculate the corresponding amino acid concentrations in the affinity purified samples (Class-VP v5.03, Shimadzu, Japan).

### Mass spectrometry

The samples after CIEX chromatography were S-alkylated with iodacetamide and digested in-solution with trypsin (Promega, WI, USA). The digested samples were loaded on an ACQUITY PRM HSST 3.1 C18 column (1.8 µm, 2.1 × 150 mm, Waters, MA, USA). 0.1% formic acid was used as aqueous solvent (A) and a mixture of 80% Acetonitrile and 20% A was used as elution buffer (B). A gradient from 3.5% B to 40% B in 35 min was applied and followed by a gradient from 40% B to 95% B in 5 min at a flow rate of 6 µL/min. The second gradient should facilitate the elution of large peptides. An Agilent Series 6560 LC-IMS-Q-TOF–MS instrument (Agilent, CA, USA) with the Jetstream ESI source in positive ion, switching to MS–MS mode for eluting peaks (DDA mode) was used for detection. MS-scans were recorded from 400 – 3200 Da and the 5 highest peaks were selected for fragmentation. Calibration was done using ESI calibration mixture (Agilent, CA, USA).

Norleucine was quantified by using the software Skyline (version 21.2) and integration of the extracted-ion chromatograms of the first four isotopic peaks of the peptides. Additionally, the analysis files were converted to mgf files for further analysis using MS/MS ion search with X!-Tandem. The files were searched against an *E. coli* database, additionally containing the sequence of the used Fab FTN2.

## Supplementary Information

**Additional file 1: Figure S1**. Fab fragment expression patterns obtained via western blot analysis for comparison of the soluble and IB fractions of different cultivations. **Table S1**. Loading scheme for the western blots shown in Figure S1. **Table S2.** Extracellular DNA content at fermentation end. **Figure S2.** Elution profiles for ProteinG affinity chromatography (a) and CIEX chromatography (b). **Figure S3.** Elution profiles of the HPLC amino acid quantification. (a) shows a low norleucin sample (B<oFTN2>, Reference, at fermentation end) and (b) shows a high norleucin sample (H<oFTN2>, Scale-down, fermentation end).

## Data Availability

The datasets generated or analyzed during the current study are available from the corresponding author on reasonable request.

## Abbreviations

CDM: Cell dry mass
CIEX: Cation exchange
DO: Dissolved oxygen
E. coli: Escherichia coli
ELISA: Enzyme linked immunosorbent assay
Fab: Antigen binding fragment
h: Hours
HC: Fab heavy chain
HPLC: High performance liquid chromatography
IB: Inclusion body
LC: Fab light chain
µ: Growth rate [h.−1]
MP: Measurement point
MS: Mass spectrometry
ncAA: Non-canonical amino acid
OmpC: Outer membrane porin C
PFR: Plug-flow reactor
POI: Protein of interest
RT: Residence time
sec: Seconds
STR: Stirred tank reactor

## References

[1] Walsh G (2018). Biopharmaceutical benchmarks 2018. Nat Biotechnol.

[2] Huang C-J, Lin H, Yang X (2012). Industrial production of recombinant therapeutics in *Escherichia coli* and its recent advancements. J Ind Microbiol Biotechnol.

[3] Rasko DA, Webster DR, Sahl JW, Bashir A, Boisen N, Scheutz F (2011). Origins of the E. coli strain causing an outbreak of hemolytic-uremic syndrome in Germany. New England J Med..

[4] Rechtsinformationssystem des Bundes - RIS. Verordnung biologische Arbeitsstoffe – VbA, Vienna, Austria. https://www.ris.bka.gv.at. Accessed 08 June 2022.

[5] Marisch K, Bayer K, Scharl T, Mairhofer J, Krempl PM, Hummel K (2013). A comparative analysis of industrial *Escherichia coli* K–12 and B strains in high-glucose batch cultivations on process-, transcriptome-and proteome level. PLoS ONE.

[6] Marisch K, Bayer K, Cserjan-Puschmann M, Luchner M, Striedner G (2013). Evaluation of three industrial *Escherichia coli* strains in fed-batch cultivations during high-level SOD protein production. Microb Cell Fact.

[7] Yoon SH, Han M-J, Jeong H, Lee CH, Xia X-X, Lee D-H (2012). Comparative multi-omics systems analysis of *Escherichia coli* strains B and K-12. Genome Biol.

[8] Jeong H, Barbe V, Lee CH, Vallenet D, Yu DS, Choi S-H (2009). Genome Sequences of *Escherichia coli* B strains REL606 and BL21(DE3). J Mol Biol.

[9] Hausjell J, Weissensteiner J, Molitor C, Halbwirth H, Spadiut OE (2018). coli HMS174(DE3) is a sustainable alternative to BL21(DE3). Microb Cell Fact.

[10] Lara AR, Galindo E, Ramírez OT, Palomares LA (2006). Living with heterogeneities in bioreactors. Mol Biotechnol.

[11] Bylund F, Collet E, Enfors SO, Larsson G (1998). Substrate gradient formation in the large-scale bioreactor lowers cell yield and increases by-product formation. Bioprocess Eng.

[12] Oosterhuis NMG, Kossen NWF (1984). Dissolved oxygen concentration profiles in a production-scale bioreactor. Biotechnol Bioeng.

[13] Enfors S-O, Jahic M, Rozkov A, Xu B, Hecker M, Jürgen B (2001). Physiological responses to mixing in large scale bioreactors. J Biotechnol.

[14] Reitz C, Fan Q, Neubauer P (2018). Synthesis of non-canonical branched-chain amino acids in *Escherichia coli* and approaches to avoid their incorporation into recombinant proteins. Curr Opin Biotechnol.

[15] Bogosian G, Violand BN, Dorward-King EJ, Workman WE, Jung PE, Kane JF (1989). Biosynthesis and Incorporation into Protein of Norleucine by *Escherichia coli*. J Biol Chem.

[16] Soini J, Falschlehner C, Liedert C, Bernhardt J, Vuoristo J, Neubauer P (2008). Norvaline is accumulated after a down-shift of oxygen in *Escherichia coli* W3110. Microb Cell Fact.

[17] Kiick KL, Weberskirch R, Tirrell DA (2001). Identification of an expanded set of translationally active methionine analogues in *Escherichia coli*. FEBS Lett.

[18] Lu HS, Tsai LB, Kenney WC, Lai P-H (1988). Identification of unusual replacement of methionine by norleucine in recombinant interleukin-2 produced by E. coli. Biochem Biophys Res Commun.

[19] Apostol I, Levine J, Lippincott J, Leach J, Hess E, Glascock CB (1997). Incorporation of norvaline at leucine positions in recombinant human hemoglobin expressed in *Escherichia coli*. J Biol Chem.

[20] ICH Q6B Specifications: Test procedures and acceptance criteria for biotechnological/biological products. European Medicines Agency. 1999. https://www.ema.europa.eu/en/documents/scientific-guideline/ich-q-6-b-test-procedures-acceptance-criteria-biotechnological/biological-products-step-5_en.pdf. Accessed 08 June 2022.

[21] Inventors: Fenton D, Lai H, Lu H, Mann M, Tsai L. Assignee: Amgen Inc., Thousand Oaks, Calif. Control of norleucine incorporation into recombinant proteins. USA. Patent: 5,599,690. 1997.

[22] Inventors: Bogosian G, O'neil JP, Smith HQ. Assignee: Monsanto Technology LLC, St. Louis, MO (US). Prevention of incorporation of non-standard amino acids into protein. USA. Patent: US 8,603,781 B2. 2013.

[23] Neubauer P, Junne S (2010). Scale-down simulators for metabolic analysis of large-scale bioprocesses. Curr Opin Biotechnol.

[24] Limberg MH, Pooth V, Wiechert W, Oldiges M (2016). Plug flow versus stirred tank reactor flow characteristics in two-compartment scale-down bioreactor: Setup-specific influence on the metabolic phenotype and bioprocess performance of *Corynebacterium glutamicum*. Eng Life Sci.

[25] Sunya S, Delvigne F, Uribelarrea J-L, Molina-Jouve C, Gorret N (2012). Comparison of the transient responses of *Escherichia coli* to a glucose pulse of various intensities. Appl Microbiol Biotechnol.

[26] Junne S, Klingner A, Kabisch J, Schweder T, Neubauer P (2011). A two-compartment bioreactor system made of commercial parts for bioprocess scale-down studies: impact of oscillations on *Bacillus subtilis* fed-batch cultivations. Biotechnol J.

[27] Mayer F, Cserjan-Puschmann M, Haslinger B, Shpylovyi A, Sam C, Soos M (2022). Using computational fluid dynamics simulation improves the design and subsequent characterization of a plug-flow type scale-down reactor for microbial cultivation processes. Authorea.

[28] Phue J-N, Noronha SB, Hattacharyya R, Wolfe AJ, Shiloach J (2005). Glucose metabolism at high density growth of E. coli B and E. coli K: differences in metabolic pathways are responsible for efficient glucose utilization in E. coli B as determined by microarrays and Northern blot analyses. Biotechno Bioeng.

[29] Spadiut O, Capone S, Krainer F, Glieder A, Herwig C (2014). Microbials for the production of monoclonal antibodies and antibody fragments. Trends Biotechnol.

[30] de Marco A (2011). Biotechnological applications of recombinant single-domain antibody fragments. Microb Cell Fact.

[31] Fink M, Cserjan-Puschmann M, Reinisch D, Striedner G (2021). High-throughput microbioreactor provides a capable tool for early stage bioprocess development. Sci Rep.

[32] Brognaux A, Francis F, Twizere J-C, Thonart P, Delvigne F (2014). Scale-down effect on the extracellular proteome of *Escherichia coli*: correlation with membrane permeability and modulation according to substrate heterogeneities. Bioprocess Biosyst Eng.

[33] Hewitt CJ, Onyeaka H, Lewis G, Taylor IW, Nienow AW (2007). A comparison of high cell density fed-batch fermentations involving both induced and non-induced recombinant *Escherichia coli* under well-mixed small-scale and simulated poorly mixed large-scale conditions. Biotechnol Bioeng.

[34] Ni J, Gao M, James A, Yao J, Yuan T, Carpick B (2015). Investigation into the misincorporation of norleucine into a recombinant protein vaccine candidate. J Ind Microbiol Biotechnol.

[35] Shiloach J, Kaufman J, Guillard AS, Fass R (1996). Effect of glucose supply strategy on acetate accumulation, growth, and recombinant protein production by Escherichia coli BL21 (λDE3) and *Escherichia coli* JM109. Biotechnol Bioeng.

[36] Noronha SB, Yeh HJC, Spande TF, Shiloach J (2000). Investigation of the TCA cycle and the glyoxylate shunt in *Escherichia coli* BL21 and JM109 using 13C-NMR/MS. Biotechnol Bioeng.

[37] Veeravalli K, Laird MW, Fedesco M, Zhang Y, Yu XC (2015). Strain engineering to prevent norleucine incorporation during recombinant protein production in *Escherichia coli*. Biotechnol Prog.

[38] Ragionieri L, Vitorino R, Frommlet J, Oliveira JL, Gaspar P, Pouplana L (2015). Improving the accuracy of recombinant protein production through integration of bioinformatics, statistical and mass spectrometry methodologies. The FEBS J.

[39] Fink M, Vazulka S, Egger E, Jarmer J, Grabherr R, Cserjan-Puschmann M (2019). Microbioreactor cultivations of fab-producing escherichia coli reveal genome-integrated systems as suitable for prospective studies on direct fab expression effects. Biotechnol J.

[40] Schimek C, Kubek M, Scheich D, Fink M, Brocard C, Striedner G (2021). Three-dimensional chromatography for purification and characterization of antibody fragments and related impurities from *Escherichia coli* crude extracts. J Chromatogr A.

[41] Altmann F (1992). Determination of amino sugars and amino acids in glycoconjugates using precolumn derivatization with o-phthalaldehyde. Anal Biochem.

