# Supplementary material for: Strain specific properties of Escherichia coli can prevent non-canonical amino acid misincorporation caused by scale-related process heterogeneities
Source: Microb Cell Fact. 2022 Aug 23;21:170. doi: 10.1186/s12934-022-01895-1 (PMC9396823; doi:10.1186/s12934-022-01895-1)
Supplement: Supplementary file 1 — Additional file 1: Figure S1. Fab fragment expression patterns obtained via western blot analysis for comparison of the soluble and IB fractions of different cultivations. Table S1. Loading scheme for the western blots shown in Figure S1. Table S2. Extracellular DNA content at fermentation end. Figure S2. Elution profiles for ProteinG affinity chromatography (a) and CIEX chromatography (b). Figure S3. Elution profiles of the HPLC amino acid quantification. (a) shows a low norleucin sample (B, Reference, at fermentation end) and (b) shows a high norleucin sample (H, Scale-down, fermentation end). [file 12934_2022_1895_MOESM1_ESM.pdf]

Supplementary Information

Solubilization of the IB fraction, as well as the western blots were done as described by Fink *et al.* [39]. Anti-human κ-LC (bound and free) goat antibody, conjugated to alkaline phosphatase (A3813; Sigma-Aldrich) was used for detection. For the molecular weight standard (PageRuler^{TM} prestained protein ladder, Thermo Fisher Scientific) 8 µL and for the other samples 15 µL were loaded. The loading scheme can be found in Table S1.

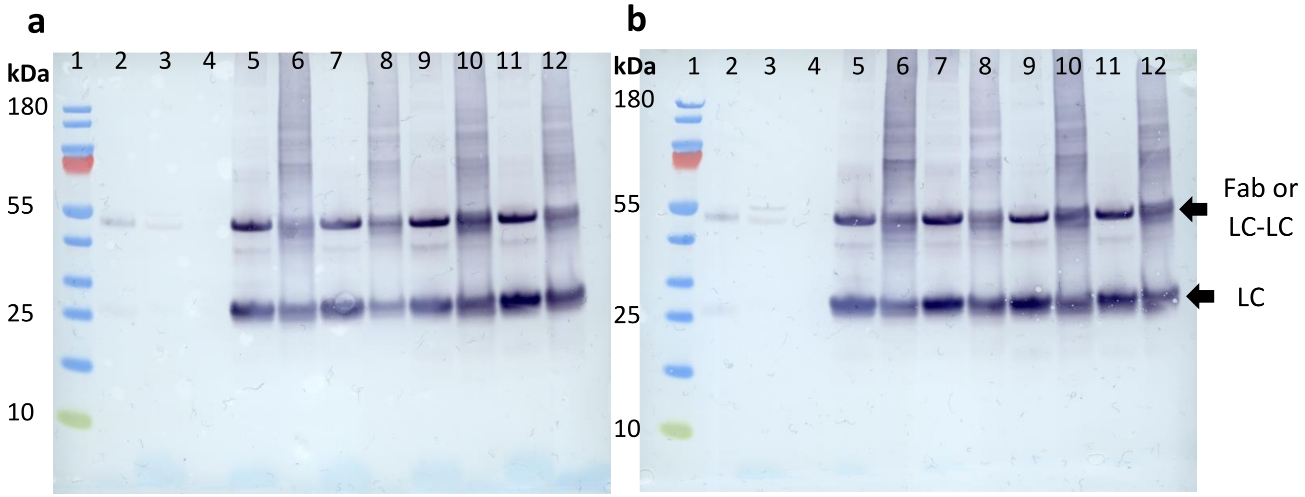

Figure S1: Fab fragment expression patterns obtained via western blot analysis for comparison of the soluble and IB fractions of different cultivations. Equal biomass amounts were applied for all samples. The loading scheme can be found in Table S1.

Table S1: Loading scheme for the western blots shown in Figure S1.

Well	Loading of blot a	Loading of blot b
1	Molecular weight standard	Molecular weight standard
2	Fab Standard (5 µg/mL)	Fab Standard (5 µg/mL)
3	B<oFTN2>_19 h (non-induced), reference soluble, 1st replicate	B<oFTN2>_19 h (non-induced), reference, soluble, 2nd replicate
4	B<oFTN2>_19 h (non-induced), reference, IB, 1st replicate	B<oFTN2>_19 h (non-induced), reference IB, 2nd replicate
5	B<oFTN2>_28.5h, reference, soluble, 1st replicate	B<oFTN2>_28.5h, reference, soluble, 2nd replicate
6	B<oFTN2>_28.5h, reference, IB, 1st replicate	B<oFTN2>_28.5h, reference, IB, 2nd replicate
7	B<oFTN2>_28.5h, scale-down, soluble, 1st replicate	B<oFTN2>_28.5h, scale-down, soluble, 2nd replicate
8	B<oFTN2>_28.5h, scale-down, IB, 1st replicate	B<oFTN2>_28.5h, scale-down, IB, 2nd replicate
9	H<oFTN2>_29h, reference, soluble, 1st replicate	H<oFTN2>_29h, reference, soluble, 2nd replicate
10	H<oFTN2>_29h, reference, IB, 1st replicate	H<oFTN2>_29h, reference, IB, 2nd replicate
11	H<oFTN2>_29h, scale-down, soluble, 1st replicate	H<oFTN2>_29h, scale-down, soluble, 2nd replicate
12	H<oFTN2>, scale-down, IB, 1st replicate	H<oFTN2>, scale-down, IB, 2nd replicate

Extracellular DNA content from the cell supernatant was measured at fermentation end, by using Qubit dsDNA BR Assay kit (Thermo Fisher Scientific, MA, USA) and Qubit fluorometer (Thermo Fisher Scientific, MA, USA) according to the manufacturer's instructions.

Table S2: Extracellular DNA content at fermentation end. The DNA concentrations are given as mean of biological duplicates. The column 'Deviation to average' gives the deviation of the individual experiments to the mean.

Experiment	DNA concentration [µg/mL]	Deviation to average [µg/mL]
B<oFTN2> Reference	1643	± 28
B<oFTN2> Scale-down	482	± 30
H<oFTN2> Reference	375	± 16
H<oFTN2> Scale-down	335	± 46

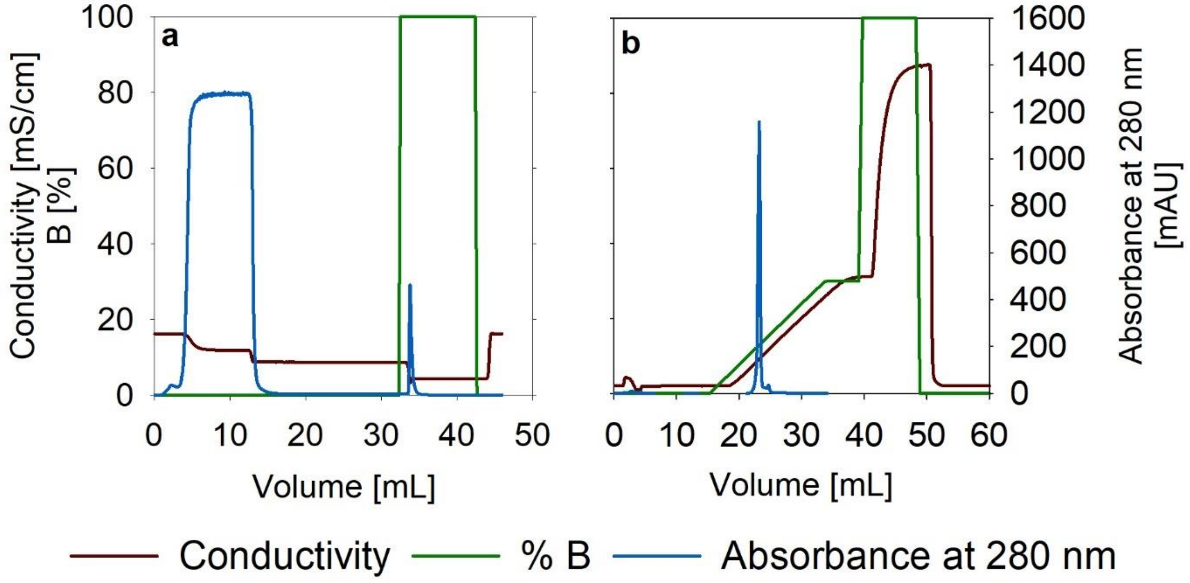

Figure S2: Elution profiles for ProteinG affinity chromatography (a) and CIEX chromatography (b). The peaks were sampled as long as the absorbance was higher than 20 mAU for the ProteinG affinity chromatography and higher than 10 mAU for the CIEX chromatography.

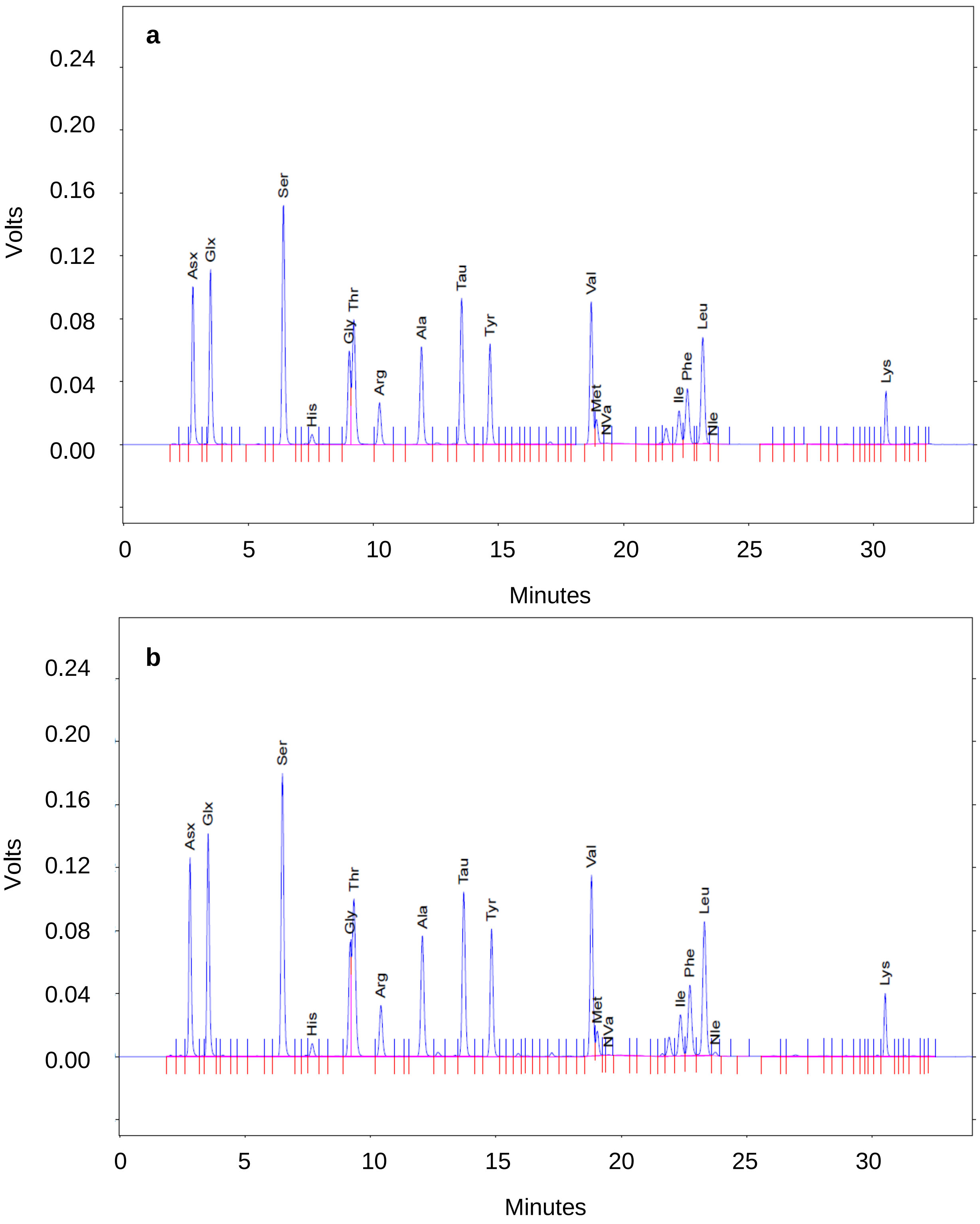

Figure S3: Elution profiles of the HPLC amino acid quantification. (a) shows a low norleucin sample (B<oFTN2>, Reference, at fermentation end) and (b) shows a high norleucin sample (H<oFTN2>, Scale-down, fermentation end)

Table S3: Empirical correction factors used for amino acid quantification with the taurine standard.

Amino acid	Correction factors [-]
Asx	0.836
Glx	0.771
Ser	0.905
His	0.715
Gly	0.588
Thr	0.938
Arg	0.898
Ala	0.784
Tyr	0.900
Val	0.993
Met	0.842
Nva	0.913
Ile	1.058
Phe	0.926
Leu	0.888
Nle	0.764
Lys	0.468
